# Cerebrospinal fluid levels of tumour necrosis factor-*α* and its receptors are not associated with disease progression in Alzheimer’s disease

**DOI:** 10.3389/fnagi.2025.1547185

**Published:** 2025-04-14

**Authors:** Manal Aljuhani

**Affiliations:** Radiological Science and Medical Imaging Department, College of Applied Medical Sciences, Prince Sattam Bin Abdulaziz University, Al-Kharj, Saudi Arabia

**Keywords:** Alzheimer’s disease, Alzheimer’s Disease Neuroimaging Initiative, biomarker, cerebrospinal fluid, proinflammatory, tumour necrosis factor

## Abstract

**Introduction:**

Tumour necrosis factor-*α* (TNF-*α*) is a proinflammatory cytokine implicated in the regulation of innate and adaptive immunity. Two receptors exist for TNF-*α*: TNF receptors 1 (TNFR-1) and 2 (TNFR-2). TNFR-1 and TNFR-2 have been reported to be involved in pleiotropic functions. Multiple lines of evidence implicate TNF-*α* and its receptors as potential risk factors for Alzheimer’s disease (AD). Studies are warranted to assess the association of TNF-*α*, TNFR-1, and TNFR-2 with AD pathogenesis and whether they can serve as prognostic biomarkers indicative of AD.

**Methods:**

In the present study, baseline levels of cerebrospinal fluid (CSF) TNF-*α*, TNFR-1, and TNFR-2 were explored, and their potential as biomarkers to differentiate between individuals who remain stable and those who experience disease progression over 10 years in the Alzheimer’s Disease Neuroimaging Initiative (ADNI) was assessed. The study also examined the correlation between baseline CSF proteins with established AD biomarkers, neuroimaging measures, and cognition.

**Results:**

Whilst the present study shows associations between baseline CSF levels of TNFs with AD biomarkers, the nature of the relationship is ambiguous.

**Discussion:**

The present study concludes that CSF TNFs do not serve as reliable or robust disease biomarkers of AD.

## 1 Introduction

Tumour necrosis factor-*α* (TNF-α) is a proinflammatory cytokine implicated in the regulation of innate and adaptive immunity (Fischer and Maier, 2015). TNF-*α* can trigger the production of interleukins (IL) 1, 6, and 8, which may accentuate inflammation. TNF-*α* activates astrocytes, leading to increased amyloid precursor protein and β-site amyloid precursor protein cleaving enzyme 1 expression, *γ*-secretase activation, and β-amyloid (Aβ) peptide release (Zhao et al., 2011; Blasko et al., 1999; Liao et al., 2004). Persistent brain inflammation creates a self-amplifying cycle that maintains high TNF-*α* concentrations, which contributes to the stimulation of Aβ production, promotes neuronal degeneration, and reduces microglial Aβ clearance (Koenigsknecht-Talboo and Landreth, 2005). The role of TNF-*α* in exacerbating tau hyperphosphorylation is less clear, but some suggestive data, particularly preclinical, point to a potential link (Montgomery et al., 2013; Plantone et al., 2023). The innate immune receptor triggering receptor expressed on myeloid cells 2 (TREM2) can modulate TNF-*α* release through the Toll-receptor pathway. Altered TREM2 functionality in monocytes or macrophages may lead to systemic TNF-*α* production, which may serve as a potential risk factor for AD that is amenable to treatment (Li et al., 2022). Specific genetic polymorphisms of TNF-α have been associated with an increased risk of AD. For example, −850 C > T polymorphism and −308 A/G polymorphism have been associated with increased AD risk (Di Bona et al., 2009; Yang et al., 2009).

Two receptors exist for TNF-*α*: TNF receptors 1 (TNFR-1) and 2 (TNFR-2). TNFR-1 and TNFR-2 have been reported to be involved in pleiotropic functions (Grell et al., 1995; Orti-Casan et al., 2019). TNFR-1 and TNFR-2 can work in cooperation to augment neuroprotection, including cell survival via nuclear factor kappa-light-chain-enhancer of activated B cells (NFκB), indicative of the pleiotropic roles played by the TNF family (Plantone et al., 2023). Studies are warranted to assess the association of TNF-*α*, TNFR-1, and TNFR-2 with AD pathogenesis and whether they can serve as prognostic biomarkers indicative of AD.

TNFR-1 and TNFR-2 are shed from the cell surface by TACE/ADAM17 (transmembrane disintegrin metalloprotease) into soluble forms that can be detected in the cerebrospinal fluid (CSF). Moreover, TACE cleaves precursor TNF-*α*, leading to the secretion of soluble, secreted TNF-*α* (Jiang et al., 2011).

In the present study, baseline levels of CSF TNF-*α*, TNFR-1, and TNFR-2 were explored, and the potential to be used as a biomarker was assessed to differentiate between individuals who remain stable compared to those who experience disease progression over 10 years in the Alzheimer’s Disease Neuroimaging Initiative (ADNI). The study also examined the correlation between baseline CSF proteins with established AD biomarkers, neuroimaging measures, and cognition. Whilst the present study showed associations between baseline CSF levels of TNFs with AD biomarkers, the nature of the relationship is ambiguous. The present study concludes that CSF TNFs do not serve as reliable or robust disease biomarkers of AD.

## 2 Materials and methods

### 2.1 ADNI study

Data used in the preparation of this article were downloaded on September 2024 from the ADNI database^1^—ADNI 1 and ADNI GO. The ADNI study has previously been described in detail (Weiner et al., 2012). The patients/participants provided their written informed consent to participate in this study. All the participants underwent apolipoprotein E (APOE) ε4 genotyping. ADNI uses serial clinical and neuropsychological assessments, imaging, and CSF biomarkers to monitor disease progression. Written informed consent was obtained and approved by the institutional review board at the participating centres for the use of human data in the ADNI database. The studies involving human participants were reviewed and approved as per ADNI protocols. All procedures performed in studies involving human participants were in accordance with the ethical standards of the institutional and/or national research committee and with the 1964 Helsinki Declaration and its later amendments or comparable ethical standards. More details can be found at http://adni.loni.usc.edu/. Data used in the preparation of this article are available in the ADNI database (see text footnote 1).

ADNI was launched in 2003 as a $60 million, 5-year public–private partnership by the National Institute on Aging (NIA), the National Institute of Biomedical Imaging and Bioengineering (NIBIB), the Food and Drug Administration (FDA), private pharmaceutical companies, and non-profit organizations, The primary goal of ADNI has been to test whether serial magnetic resonance imaging (MRI), PET, other biological markers, and clinical and neuropsychological assessments can be combined to monitor disease progression. Determination of sensitive and specific markers of very early AD progression aids researchers/clinicians in developing new treatments, monitoring their effectiveness, and identifying suitable participants for enrollment in clinical trials. The principal investigator of this initiative is Michael W. Weiner, MD, VA Medical Centre and University of California, San Francisco, supported by many co-investigators from a broad range of academic institutions and private corporations. Subjects have been recruited from over 50 sites across the US and Canada. For up-to-date information, please see http://www.adni-info.org/.

### 2.2 CSF measurements for TNF-*α*, TNFR-1, and TNFR-2

CSF analysis was performed by two skilled research scientists experienced in multiplex assays blinded to diagnosis and other subject-level information. All samples were run in duplicate with six CSF standards on each plate, and CSF inflammatory protein levels were normalized across plates using the six CSF standard values. ADNI CSF samples were first randomized across twelve 96-well plates, and each batch was analyzed for levels of proteins during the same 2-day block to avoid freeze–thawing. TNF-*α*-related inflammatory proteins were obtained and measured at Emory University using ELISA kits. All samples were run in duplicate with six CSF standards on each plate. Samples were normalized across plates using CSF standard values. All samples were run in duplicate with six CSF standards on each plate. CSF inflammatory protein levels were normalized across plates using the six CSF standard values, and intermediate precision for each analyte was then calculated using the interplate coefficient of variation (Table 1).

**Table 1:** Summary of data and precision of each analyte for TNF-α, TNFR-1, and TNFR-2 across plates expressed as percent coefficient of variation.

CSF proteins	Standard range (pg/mL)	Median sample concentration (pg/mL)	LLOD (MFI)	Median MFI	Interplate %CV
TNF-α	1.6–10,000	1.70	49.2	53.53	9.38
TNFR-1	12.2–50,000	854.44	28.10	1384.13	2.85
TNFR-2	12.2–50,000	1013.88	16.39	1474.13	3.09

CV, coefficient of variation; LLOD, lower limit of detection; MFI, mean fluorescence intensity; TNF, tumour necrosis factor; TNFR, tumour necrosis factor receptor.

### 2.3 CSF measurements for Aβ, total tau, and p-tau

CSF samples were obtained in the morning following overnight fasting at the baseline visit. The time from collection to freezing was ~1 h, with processing, aliquoting, and storage at −80°C as per ADNI Biomarker Core Laboratory Standard Operating Procedures. CSF Aβ1–42, total tau, and p-tau were measured using the Luminex platform as described previously (Jagust et al., 2009; Shaw et al., 2011).

### 2.4 Inclusion/exclusion criteria

Enrolled subjects in the ADNI-1 cohort were 55–90 years of age, accompanied by a study partner able to provide an independent evaluation of the recruited participant’s functioning and speak either English or Spanish fluently. Participants must have a Hachinski Ischemic score ≤ 4, geriatric depression scale <6, visual and auditory acuity adequate for neuropsychological testing, six grades of education or work history, and not be enrolled in other trials or studies. Individuals on specific psychoactive medications, e.g., narcotic analgesics, neuroleptics, anticholinergic agents, antiparkinsonian medications, investigational drugs, benzodiazepines, antihypertensive agents with frequent central nervous system side effects, and antidepressants, within 4 weeks prior to screening were excluded. Individuals with any serious neurological disease other than AD, or any history of brain lesions or brain trauma were also excluded.

Cognitively normal (CN) subjects must have no significant impairment in their cognitive domains or impaired activities of daily living, with a Mini-Mental State Examination (MMSE) score between 24 and 30, a CDR of 0, no depression, no MCI, and no dementia. The age range of CN individuals was matched to that of MCI and AD subjects. MCI subjects must also have an MMSE score between 24 and 30 but have a memory complaint, experienced objective memory loss as measured by education-adjusted scores on the Wechsler Memory Scale Logical Memory II, a CDR of 0.5, although no significant impairment in other cognitive domains, and preserved activities of daily living, and be free of dementia. The AD cases included in the study had MMSE scores between 20 and 26, a CDR of 0.5 or 1.0, and met the National Institute of Neurological and Communicative Disorders and Stroke-Alzheimer’s Disease and Related Disorders Association criteria for probable AD (NINDS-ADRDA).

### 2.5 Tracking disease progression

Tracking disease progression is a primary outcome measure of the ADNI protocol. Site physicians review participants’ data and complete diagnostic summaries. If a physician triggers a change in diagnosis, e.g., from CN to MCI or MCI to AD, the clinical monitor onsite reviews the neuropsychological assessments for that visit. The clinical monitor will resolve any issues with the site’s primary investigator and instruct the diagnosis to be reversed if incorrectly reported. An ADNI clinical co-investigator subsequently reviews the data and requests the clinical monitor to resolve any scoring issues. When this review is finalized, the ADNI conversion committee is then commissioned with the task of reviewing all patient reports, and a consensus on the conversion status of the participant is achieved as per the NINDS-ARCDA (please refer to the general procedures manual; http://adni.loni.usc.edu/). Although a neuropathological diagnosis is required to confirm the diagnosis of AD, studies have shown high sensitivity and specificity using neuroimaging and neuropsychological assessments for determining disease progression to AD (Petersen et al., 2010; Davatzikos et al., 2001; Fan et al., 2008).

### 2.6 Structural MRI volumes

Subjects underwent structural MRI at 1.5 T using a 3D sagittal volumetric magnetization-prepared rapid gradient echo (MP-RAGE) sequence (Jack et al., 2010). The acquisition parameters were repetition time, 9 ms; echo time, 4 ms; flip angle 8°, with a 256 × 256 × 170 acquisition matrix in the x-, y-, and z-dimensions with a nominal voxel size of 0.94 × 0.94 × 1.2 mm^3^. MRI was performed at baseline, 6 months, 1 year, and then yearly for 6 years. FreeSurfer (version 4.1.0) was used to calculate hippocampal volumes and was described previously (Fischl et al., 2002). Briefly, MRI volumetric images initially underwent motion correction, hybrid watershed or surface deformation removal of non-brain tissue, automated Talairach transformation, and then the segmentation of subcortical white and deep grey matter structures. This was followed by intensity normalization, tessellation of the grey and white matter boundary, and automated topology correction. The hippocampus and amygdala have similar signal intensities, but their spatial location is quite consistent relative to one another; the amygdala is always in front of and above the hippocampus. To ensure the segmentations were anatomically plausible, the Markov random field model was used and modified to be spatially non-stationary. This involved separately modelling the probabilities of the hippocampus above and below the amygdala, resulting in accurate identification of the hippocampus. Hippocampal volume was calculated by multiplying the number of hippocampal voxels by the voxel volume.

### 2.7 [^18^F] Fluorodeoxyglucose ([^18^F]FDG-PET)

[^18^F]FDG-PET scans were acquired on multiple scanners at various resolutions at 6 months, 1, 1.5, and 2 years (Jagust et al., 2010). The scans were acquired as 6 × 5-min frames, for 30 min after injection of 5 mCi of ^18^F-FDG. The FDG-PET images were pre-processed according to standard ADNI procedures with frames co-registered, averaged, and reoriented along the anterior–posterior commissure line and resliced to a 1.5 mm isotropic voxel space. Each PET image was spatially normalized to Montreal Neurological Institute (MNI) space, and the mean hippocampal FDG uptake (normalized to pons uptake) was measured.

### 2.8 Neuropsychological assessments

The Rey Auditory Verbal Learning Test (RAVLT) and the Alzheimer’s Disease Assessment Scale-Cognitive Subscale 13-item version (ADAS-Cog13) were used as sensitive markers of disease progression. RAVLT tests episodic verbal memory by assessing an individual’s ability to acquire a list of 15 words over five trials. The test comprised a short-delay recall trial presented after a distracter list, a 30-min long delay recall trial, and finally a yes/no recognition trial.^2^ ADAS-Cog13 is a 13-item scale used for assessing learning, memory, language production and comprehension, constructional and ideational praxis, orientation, number cancellation, and delayed free recall tasks. The word recall test was administered first, and the word recognition task was given at the end with other cognitive tasks given in between. The two-word memory tasks were separated so that the risk of individuals confusing words from the two tasks was minimized. Objective testing was followed by subjective clinical ratings of the language ability and aptitude of the participant to remember test instructions (extended details can be found on the ADNI website: adni.loni.usc.edu/wp-content/uploads/2010/09/ADNI_GeneralProceduresManual.pdf).

### 2.9 Statistical analysis

To understand how CSF cytokines/receptors are affected at different stages of the disease and whether the trend observed in proteins is reproducible and consistent across cohorts, we divided the ADNI dataset into exploratory cohorts (cohorts 1–2), a protein threshold-defining dataset comprised of CN individuals, and the main cohort:

- Exploratory Cohort 1: baseline cohort (3 CN and 15 cognitively impaired [6 MCI and 9 AD]).
- Exploratory Cohort 2: AD baseline and longitudinal cohort (*n* = 78).
- Protein threshold-defining dataset: CN longitudinal cohort (*n* = 58; used only to formulate thresholds to label protein levels as low, medium, or level).
- Main Cohort: Individuals who remain stable (*n* = 103) and those who demonstrate disease progression (*n* = 93).

Welch’s t-test was used to examine the baseline levels of CSF cytokines/receptors (TNF-*α*, TNFR-1, and TNFR-2) in CN compared to cognitively impaired (Exploratory Cohort 1), which included MCI and AD. Next, Pearson’s correlation analyses were performed on the AD dataset (Exploratory Cohort 2) to assess the relationship of CSF cytokines/receptors with AD protein biomarkers (CSF Aβ, total tau, and p-tau), neuroimaging measures (FDG-PET [average of angular, temporal, and posterior cingulate], MRI-derived hippocampal volumes and whole brain volumes), and cognition (RAVLT and ADAS-Cog13) at baseline and over a year.

As part of the main cohort analyses, baseline levels of CSF TNF-*α*, TNFR-1, and TNFR-2 were assessed using Welch’s t-test to see if they can differentiate between individuals who remain stable compared to those that have disease progression within the next 10 years. Following this, tertile analysis was used to label the proteins as low (x < Q1), medium (Q1 ≤ x ≤ Q3), or high (x > Q3), based on protein-threshold defining Cohort, and examine their association with annual changes (to control for variable follow-up times) in AD pathology, neuroimaging and cognitive biomarkers using one-way ANOVA followed by *post hoc* Tukey correction. TNF-*α* protein levels were labelled as low (TNF-α < 1.2875), medium (1.2875 ≤ TNF-α ≤ 1.9975), or high (TNF-α > 1.9975). TNFR-1 protein levels were labelled as low (TNFR-1 < 712.79), medium (712.79 ≤ TNFR-1 ≤ 989.28), or high (TNFR-1 > 989.28). TNFR-2 protein levels were labelled as low (TNFR-2 < 793.76), medium (793.76 ≤ TNFR-2 ≤ 1129.07), or high (TNFR-2 > 1129.07).

All analyses were performed using GraphPad Prism 10.4.0. Since this was an exploratory study, a *p*-value of ≤0.05 was considered significant.

## 3 Results

### 3.1 Cohort 1: cognitively impaired and CN groups

**Figure 1:**
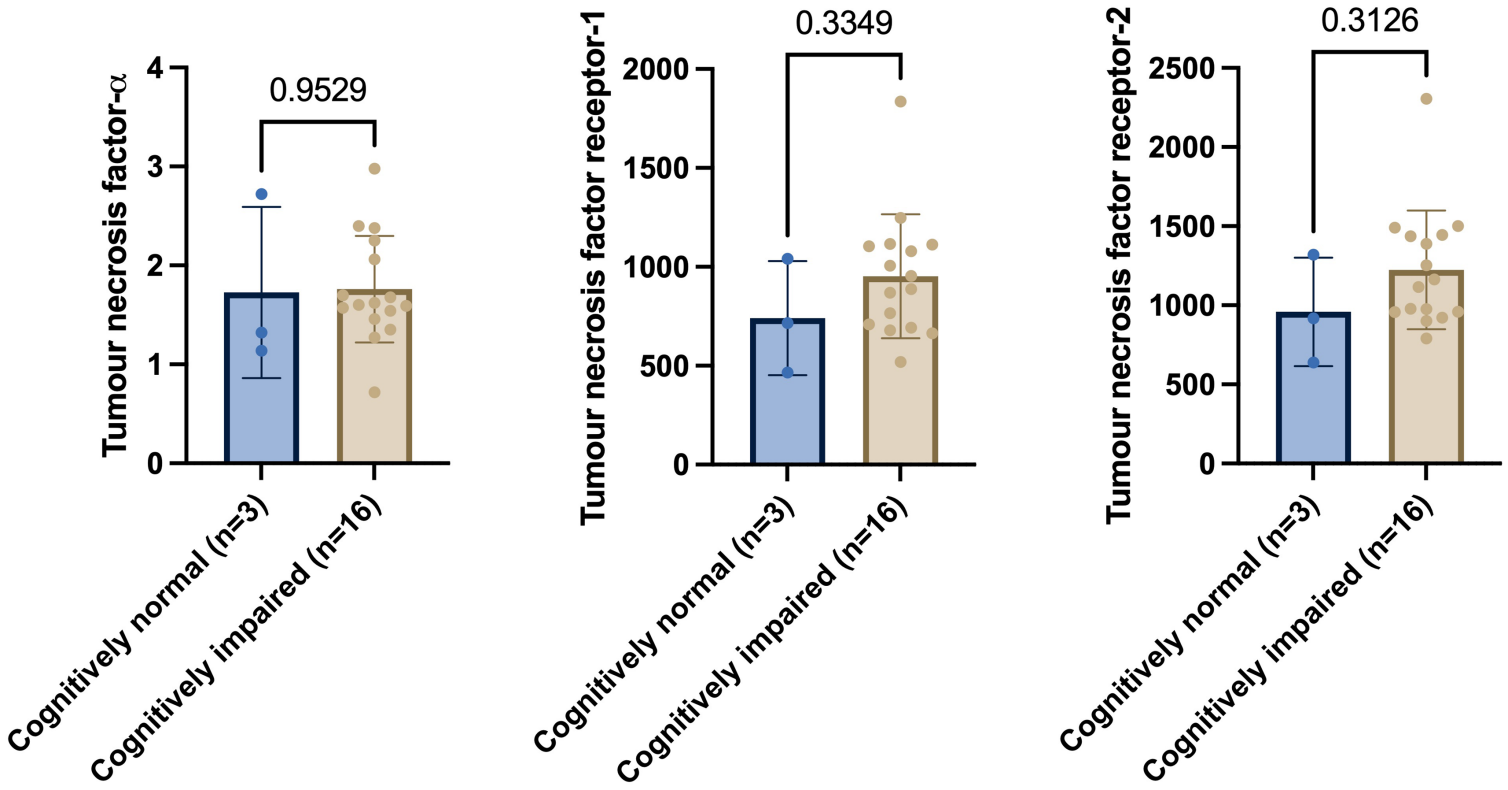
Welch’s *t*-test shows no significant differences in tumour necrosis factor-*α*, tumour necrosis factor receptor-1, and tumour necrosis receptor-2 between cognitively normal and cognitively impaired individuals. Numbers on the graph represent *p*-values. A *p*-value of ≤0.05 was considered significant.

First, CSF levels of members of the TNF family were assessed in the cognitively impaired compared to CN individuals. Welch’s t-test on exploratory cohort 1 revealed that baseline levels of TNF-*α* (*p* = 0.9529), TNFR-1 (*p* = 0.3349), and TNFR-2 (*p* = 0.3126) showed non-significant changes in cognitively impaired (MCI and AD) versus CN group (Figure 1).

### 3.2 Cohort 2: AD cohort

**Figure 2:**
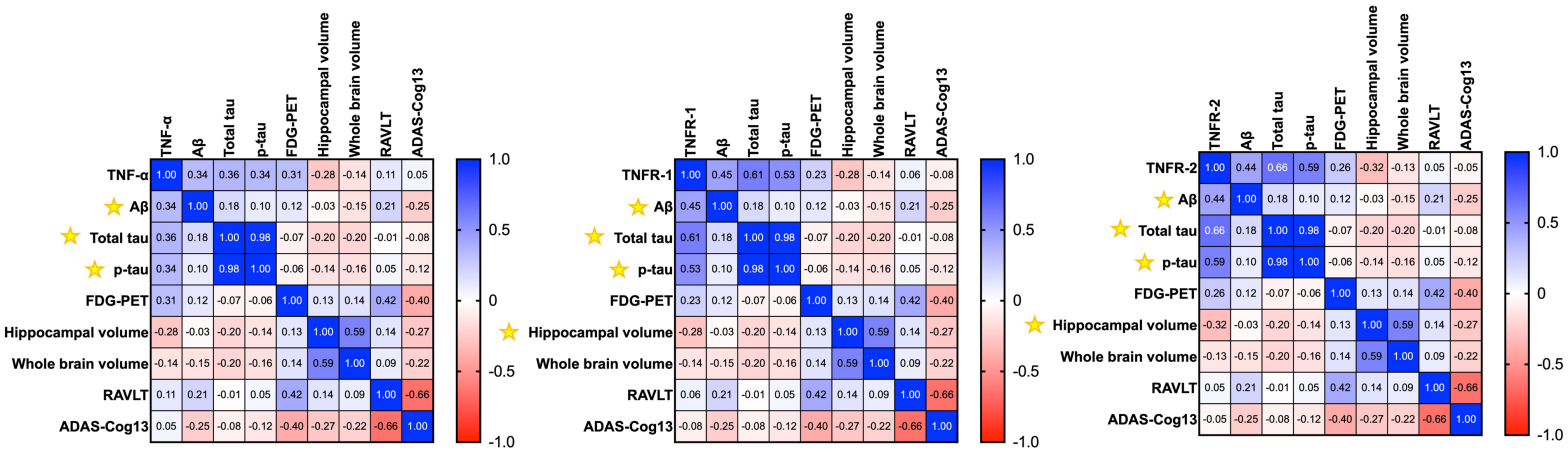
Heatmap shows the correlation matrix (Pearson’s correlation analyses) between baseline tumour necrosis factor-α (TNF-α), tumour necrosis factor receptor-1 (TNFR-1), and tumour necrosis receptor-2 (TNFR-2), with baseline Aβ, total tau, p-tau, neuroimaging measures, and cognition in patients with Alzheimer’s disease. The gradient bar shows the correlation coefficient (r). Yellow asterisk symbols denote significant associations of TNF cytokines/receptors with AD biomarkers, neuroimaging measures, and cognition. A *p*-value of ≤0.05 was considered significant.

**Figure 3:**
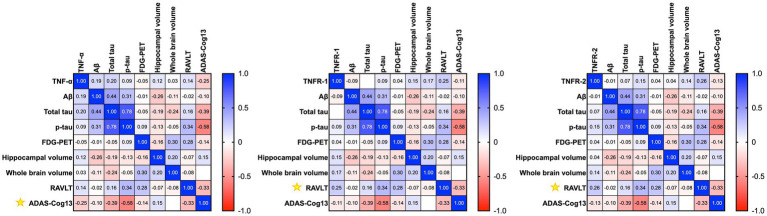
Heatmap shows the correlation matrix (Pearson’s correlation analyses) between baseline tumour necrosis factor-α (TNF-α), tumour necrosis factor receptor-1 (TNFR-1), and tumour necrosis receptor-2 (TNFR-2), with longitudinal changes in Aβ, total tau, p-tau, neuroimaging measures, and cognition after a year in patients with Alzheimer’s disease. Yellow asterisk symbols denote significant associations of TNF cytokines/receptors with AD biomarkers, neuroimaging measures, and cognition. A *p*-value of ≤0.05 was considered significant.

We wanted to assess the association between CSF levels of TNFs with baseline (Figure 2) and longitudinal changes (Figure 3) in CSF AD proteins, neuroimaging measures, and cognition in individuals with established AD (*n* = 78). The mean age ± standard deviation of the AD cohort was 74.86 ± 8.138 years, and education was 15.038 ± 3.002 years. There were 35 women (44.9%), 68 (87.2%) individuals were married, 55 (70.5%) had at least one copy of the APOE ε4 allele, and all patients with AD were white. Pearson’s correlation analyses on the AD dataset showed that higher baseline levels of TNF-*α* were significantly associated with baseline CSF Aβ (r = 0.3384, *p* = 0.0024), total tau (r = 0.3592, *p* = 0.0012), and p-tau (r = 0.3432, *p* = 0.0021). Higher baseline levels of TNF-*α* were associated with higher baseline glucose metabolism (r = 0.3145, *p* = 0.0512). High baseline levels of TNF-α were correlated with lower baseline levels of hippocampal volume (r = −0.2754, *p* = 0.0400; Figure 2). A similar trend was observed for TNFR-1 and TNFR-2. Higher levels of TNFR-1 were significantly associated with baseline CSF Aβ (r = 0.4523, *p* < 0.0001), total tau (r = 0.6115, *p* < 0.0001), p-tau (r = 0.5326, *p* < 0.0001), and hippocampal volume (r = −0.2798, *p* = 0.0367). Higher levels of TNFR-2 were significantly associated with baseline CSF Aβ (r = 0.4409, *p* < 0.0001), total tau (r = 0.6638, *p* < 0.0001), p-tau (r = 0.5932, *p* < 0.0001), and hippocampal volume (r = −0.3232, *p* = 0.0151).

Pearson’s correlation analyses of baseline levels of TNF-*α* found higher baseline levels of TNF-α associated with longitudinal changes in ADAS-Cog13 over a year (r = −0.2453, *p* = 0.0471) in patients with AD (Figure 3). Meanwhile, TNFR-1 (r = 0.2457, *p* = 0.0467) and TNFR-2 (r = 0.2578, *p* = 0.0367) were associated with longitudinal changes in RAVLT (Figure 3).

### 3.3 Main cohort: individuals who remain stable and those who demonstrate disease progression

The baseline levels of CSF TNF-α, TNFR-1, and TNFR-2 were assessed to differentiate between individuals who remain stable compared to those who demonstrate disease progression within a decade. There were 103 individuals who remained stable (mean age ± standard deviation = 75.04 ± 6.956 years), whilst 93 individuals showed disease progression (mean age 74.79 ± 7.207 years). The average years of education ± standard deviation was 16.14 ± 2.818 in the stable group compared to 15.72 ± 2.983 in the disease progression group. Of the 103 individuals who remained stable, 46 were female (44.6%), 96 were white (96.3%), 76 were married (73.8%), and 31 had at least one APOE ε4 allele (30.1%); whereas, of the 93 individuals who showed disease progression, 33 were female (35.5%), 86 were white (92.5%), 78 were married (83.9%), and 53 had at least one APOE ε4 allele (57.0%). The median follow-up time for individuals who remained stable was 3 years (range 1–10 years) and 4 years (range 1–10 years) in the disease progression group. In the disease progression group, the median time to disease progression was 2 years (1–10 years).

**Figure 4:**
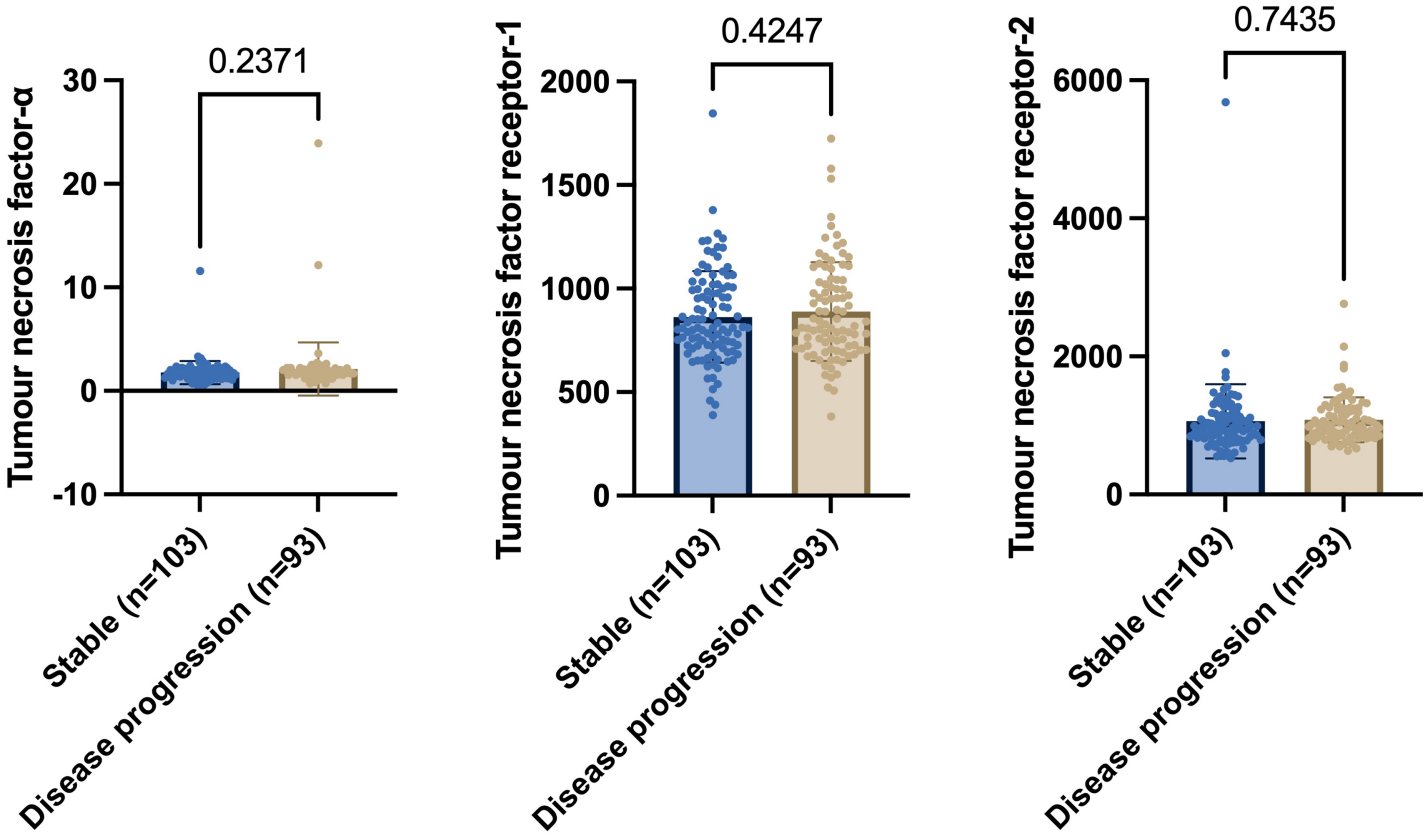
Welch’s *t*-test shows no significant differences in baseline levels of tumour necrosis factor-α, tumour necrosis factor receptor-1, and tumour necrosis receptor-2 between individuals who remain stable and those who show disease progression. Numbers on the graph represent *p*-values. A *p*-value of ≤0.05 was considered significant.

**Figure 5:**
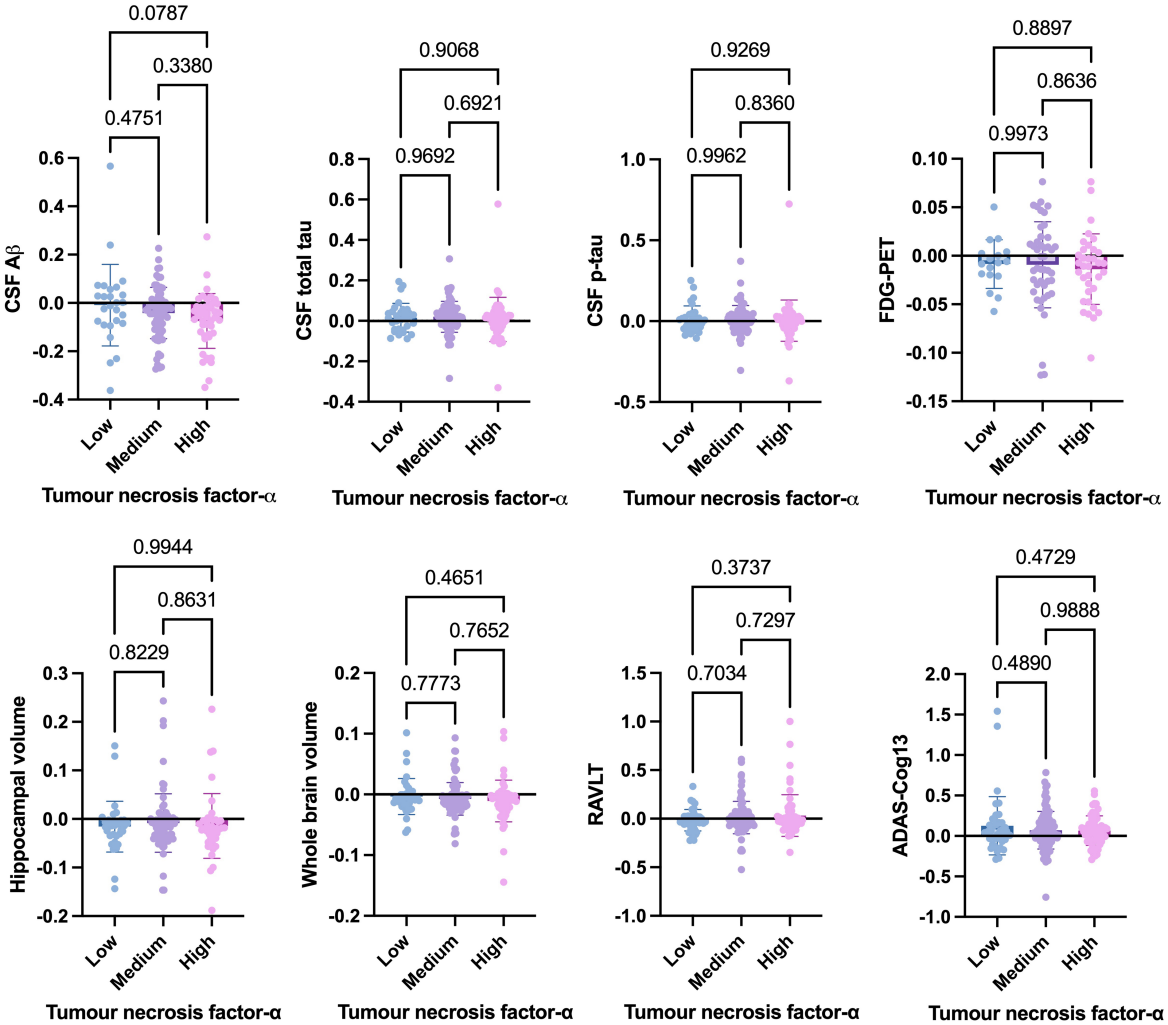
One-way ANOVA assesses annual changes in cerebrospinal fluid levels of Aβ, total tau, p-tau, neuroimaging measures, and cognition expressed as tumour necrosis factor-α tertiles [low (*n* = 39), medium (*n* = 97), and high (*n* = 60)]. Numbers on the graph represent *p*-values. A *p*-value of ≤0.05 was considered significant.

**Figure 6:**
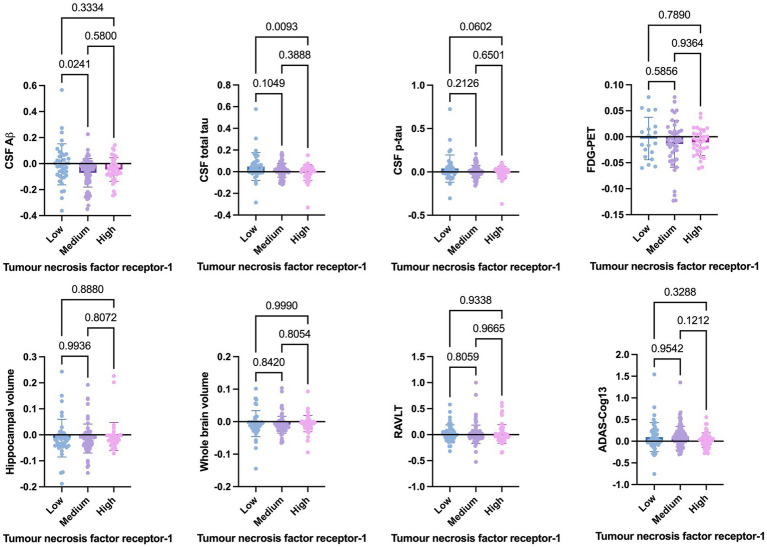
One-way ANOVA assesses annual changes in cerebrospinal fluid levels of Aβ, total tau, p-tau, neuroimaging measures, and cognition expressed as tumour necrosis factor receptor-1 tertiles [low (*n* = 39), medium (*n* = 97), and high (*n* = 60)]. Numbers on the graph represent *p*-values. A *p*-value of ≤0.05 was considered significant.

**Figure 7:**
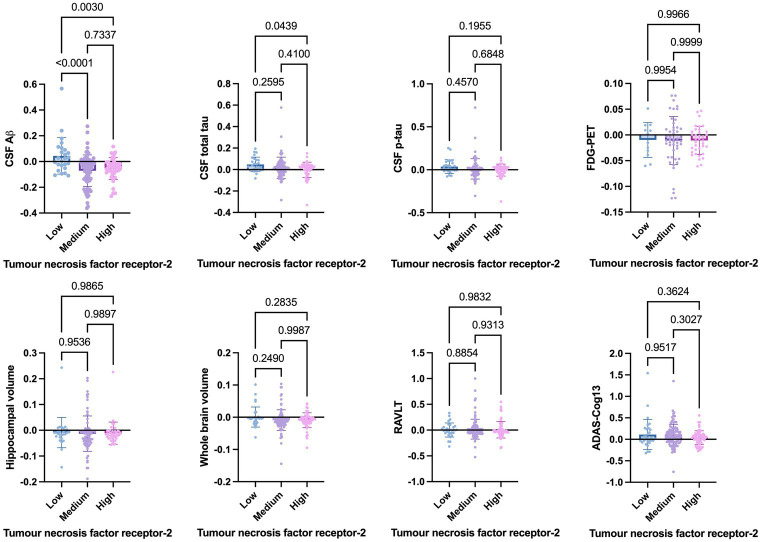
One-way ANOVA assesses annual changes in cerebrospinal fluid levels of Aβ, total tau, p-tau, neuroimaging measures, and cognition expressed as tumour necrosis factor-2 tertiles [low (*n* = 39), medium (*n* = 97), and high (*n* = 60)]. Numbers on the graph represent *p*-values. A *p*-value of ≤0.05 was considered significant.

Welch’s t-test on the main cohort revealed that the baseline levels of TNF-*α* (*p* = 0.2371), TNFR-1 (*p* = 0.4247), or TNFR-2 (*p* = 0.7435) did not significantly differentiate between individuals who remained stable versus those who had disease progression, with a follow-up of up to 10 years (Figure 4). As specified in the Methods section, the CN cohort (*n* = 58) was used to define protein levels at low, medium, or high levels for the main cohort analyses. One-way ANOVA expressed as TNF-*α* tertiles found no significant association with annual changes in AD proteins, neuroimaging measures, and cognition (Figure 5). Individuals with medium baseline levels of TNFR-1 in the main cohort had significantly decreased annual CSF Aβ compared to individuals with low baseline levels of TNFR-1 (*p* = 0.0241; Figure 6). Individuals with high baseline levels of TNFR-1 in the main cohort had significantly decreased annual CSF total tau compared to the low baseline level group (*p* = 0.0093; Figure 6). Compared to the group with low baseline levels of TNFR-2, high (*p* = 0.0030), and medium (*p* < 0.0001) baseline levels of TNFR-2 had low annual levels of CSF Aβ (Figure 7). High levels of baseline TNFR-2 had lower annual CSF total tau compared to the low baseline TNFR-2 group (*p* = 0.0439; Figure 7).

## 4 Discussion

The present study did not find significantly altered baseline CSF levels of TNF-*α*, TNFR-1, and TNFR-2 between individuals who remained stable compared to those who demonstrated disease progression. The trio was significantly associated with CSF Aβ and tau in patients at different stages of AD pathogenesis. The findings suggest that whilst the TNF family may be implicated in the disease process in AD, its utility as a disease biomarker is limited.

Although CSF studies on the TNF family are limited, the present findings of non-significant changes in TNF-α are consistent with previous studies (Tarkowski et al., 2003; Llano et al., 2012; Swardfager et al., 2010). Tarkowski et al. (2003) did not find significant differences in the initial TNF-*α* mean levels between patients who proceeded to overt AD and patients who did not at follow-up 9 months later. This suggests that TNF-α changes may not be sufficient and likely work in tandem with other cytokines/receptors and AD pathology to cause progressive deterioration. The lack of significant changes in the CSF TNF family protein levels could be due to regional variability, particularly in disease-affected brain areas, which may not lead to changes in CSF protein levels, as CSF levels reflect the summed metabolism of the whole brain. A limitation of the study is that CSF protein measurement does not assess the functional status of the proteins nor inform the protein modifications that may occur in the disease context. Studies measuring the functional status of the proteins are warranted to better understand the role of cytokines/receptors in AD pathogenesis.

The finding of a significant correlation between the TNF family and AD pathology markers is consistent with a previous study (Zhao et al., 2020), which suggests that CNS inflammation is an important event in the pathogenesis of AD. The mechanism underlying the association between the TNF family and AD pathology remains to be understood. In AD, TNF-*α* inhibition reverted the effect of tau accumulation in neurites through high-affinity binding to TNFR-1. TNFR-1 and TNFR-2 inhibition were found to exacerbate tau accumulation and neurofibrillary tangle pathology (Montgomery et al., 2013). It is likely that, TNF receptor modulation may differentially and intricately affect AD pathology at various disease stages.

A double-blind study in mild and moderate AD patients treated with subcutaneous etanercept (anti-TNF agent) did not show significant changes in cognitive function, behavior, and global functions, though there was a positive trend in the anti-TNF-α treatment group (Butchart et al., 2015). The limited effectiveness of anti-TNF-α therapies in AD patients may be attributed to the large molecular weight of anti-TNF-α monoclonal antibodies because this makes the passage through the blood–brain barrier challenging under physiological conditions. An alternative explanation supported by the present study suggests that the TNF family may not be useful disease biomarkers or is involved in disease pathogenesis but is not a significant driver of disease pathology.

Another limitation of the study is that most individuals were white, which may not be representative of broader patient populations in the real world. Therefore, independent validation is needed in a larger more heterogeneous AD dementia population to fully assess the long-term safety and clinical effects of this approach.

In conclusion, the baseline CSF levels of TNF-α and its receptors are not significantly associated with disease progression in AD.

## Data Availability

All ADNI data are available for public access at http://adni.loni.usc.edu/ contingent on adherence to the ADNI Data Use Agreement. The original contributions presented in the study are included in the article/supplementary material, further inquiries can be directed to the corresponding author.

## Footnotes

^1^ http://adni.loni.usc.edu/

^2^ http://www.adni-info.org

## References

[ref1] Blasko I. Marx F. Steiner E. Hartmann T. Grubeck-Loebenstein B. (1999). TNFalpha plus IFNgamma induce the production of Alzheimer beta-amyloid peptides and decrease the secretion of APPs. FASEB J. 13, 63–68. doi: 10.1096/fasebj.13.1.63, PMID: 9872930

[ref2] Butchart J. Brook L. Hopkins V. Teeling J. Puntener U. Culliford D.. (2015). Etanercept in Alzheimer disease: a randomized, placebo-controlled, double-blind, phase 2 trial. Neurology 84, 2161–2168. doi: 10.1212/WNL.0000000000001617, PMID: 25934853 PMC4451045

[ref3] Davatzikos C. Genc A. Xu D. Resnick S. M. (2001). Voxel-based morphometry using the RAVENS maps: methods and validation using simulated longitudinal atrophy. NeuroImage 14, 1361–1369. doi: 10.1006/nimg.2001.0937, PMID: 11707092

[ref4] Di Bona D. Candore G. Franceschi C. Licastro F. Colonna-Romano G. Camma C.. (2009). Systematic review by meta-analyses on the possible role of TNF-alpha polymorphisms in association with Alzheimer's disease. Brain Res. Rev. 61, 60–68. doi: 10.1016/j.brainresrev.2009.05.001, PMID: 19445962

[ref5] Fan Y. Batmanghelich N. Clark C. M. Davatzikos C. Alzheimer’s Disease Neuroimaging Initiative (2008). Spatial patterns of brain atrophy in MCI patients, identified via high-dimensional pattern classification, predict subsequent cognitive decline. NeuroImage 39, 1731–1743. doi: 10.1016/j.neuroimage.2007.10.031, PMID: 18053747 PMC2861339

[ref6] Fischer R. Maier O. (2015). Interrelation of oxidative stress and inflammation in neurodegenerative disease: role of TNF. Oxidative Med. Cell. Longev. 2015:610813. doi: 10.1155/2015/610813 PMC4365363 25834699

[ref7] Fischl B. Salat D. H. Busa E. Albert M. Dieterich M. Haselgrove C.. (2002). Whole brain segmentation: automated labeling of neuroanatomical structures in the human brain. Neuron 33, 341–355. doi: 10.1016/S0896-6273(02)00569-X 11832223

[ref8] Grell M. Douni E. Wajant H. Lohden M. Clauss M. Maxeiner B.. (1995). The transmembrane form of tumor necrosis factor is the prime activating ligand of the 80 kDa tumor necrosis factor receptor. Cell 83, 793–802. doi: 10.1016/0092-8674(95)90192-2, PMID: 8521496

[ref9] Jack C. R. Jr. Bernstein M. A. Borowski B. J. Gunter J. L. Fox N. C. Thompson P. M.. (2010). Update on the magnetic resonance imaging core of the Alzheimer's disease neuroimaging initiative. Alzheimers Dement. 6, 212–220. doi: 10.1016/j.jalz.2010.03.004, PMID: 20451869 PMC2886577

[ref10] Jagust W. J. Bandy D. Chen K. Foster N. L. Landau S. M. Mathis C. A.. (2010). The Alzheimer's disease neuroimaging initiative positron emission tomography core. Alzheimers Dement. 6, 221–229. doi: 10.1016/j.jalz.2010.03.003, PMID: 20451870 PMC2920531

[ref11] Jagust W. J. Landau S. M. Shaw L. M. Trojanowski J. Q. Koeppe R. A. Reiman E. M.. (2009). Relationships between biomarkers in aging and dementia. Neurology 73, 1193–1199. doi: 10.1212/WNL.0b013e3181bc010c, PMID: 19822868 PMC2764726

[ref12] Jiang H. Hampel H. Prvulovic D. Wallin A. Blennow K. Li R.. (2011). Elevated CSF levels of TACE activity and soluble TNF receptors in subjects with mild cognitive impairment and patients with Alzheimer's disease. Mol. Neurodegener. 6:69. doi: 10.1186/1750-1326-6-69 21978728 PMC3206445

[ref13] Koenigsknecht-Talboo J. Landreth G. E. (2005). Microglial phagocytosis induced by fibrillar beta-amyloid and IgGs are differentially regulated by proinflammatory cytokines. J. Neurosci. 25, 8240–8249. doi: 10.1523/JNEUROSCI.1808-05.2005, PMID: 16148231 PMC6725530

[ref14] Li R. Zhang J. Wang Q. Cheng M. Lin B. (2022). TPM1 mediates inflammation downstream of TREM2 via the PKA/CREB signaling pathway. J. Neuroinflammation 19:257. doi: 10.1186/s12974-022-02619-3 36241997 PMC9563125

[ref15] Liao Y. F. Wang B. J. Cheng H. T. Kuo L. H. Wolfe M. S. (2004). Tumor necrosis factor-alpha, interleukin-1beta, and interferon-gamma stimulate gamma-secretase-mediated cleavage of amyloid precursor protein through a JNK-dependent MAPK pathway. J. Biol. Chem. 279, 49523–49532. doi: 10.1074/jbc.M402034200, PMID: 15347683

[ref16] Llano D. A. Li J. Waring J. F. Ellis T. Devanarayan V. Witte D. G.. (2012). Cerebrospinal fluid cytokine dynamics differ between Alzheimer disease patients and elderly controls. Alzheimer Dis. Assoc. Disord. 26, 322–328. doi: 10.1097/WAD.0b013e31823b2728, PMID: 22089638

[ref17] Montgomery S. L. Narrow W. C. Mastrangelo M. A. Olschowka J. A. O'Banion M. K. Bowers W. J. (2013). Chronic neuron- and age-selective down-regulation of TNF receptor expression in triple-transgenic Alzheimer disease mice leads to significant modulation of amyloid- and tau-related pathologies. Am. J. Pathol. 182, 2285–2297. doi: 10.1016/j.ajpath.2013.02.030, PMID: 23567638 PMC3668024

[ref18] Orti-Casan N. Wu Y. Naude P. J. W. De Deyn P. P. Zuhorn I. S. Eisel U. L. M. (2019). Targeting TNFR2 as a novel therapeutic strategy for Alzheimer's disease. Front. Neurosci. 13:49. doi: 10.3389/fnins.2019.00049 30778285 PMC6369349

[ref19] Petersen R. C. Aisen P. S. Beckett L. A. Donohue M. C. Gamst A. C. Harvey D. J.. (2010). Alzheimer's disease neuroimaging initiative (ADNI): clinical characterization. Neurology 74, 201–209. doi: 10.1212/WNL.0b013e3181cb3e25, PMID: 20042704 PMC2809036

[ref20] Plantone D. Pardini M. Righi D. Manco C. Colombo B. M. De Stefano N. (2023). The role of TNF-alpha in Alzheimer's disease: a narrative review. Cells 13:54. doi: 10.3390/cells13010054, PMID: 38201258 PMC10778385

[ref21] Shaw L. M. Vanderstichele H. Knapik-Czajka M. Figurski M. Coart E. Blennow K.. (2011). Qualification of the analytical and clinical performance of CSF biomarker analyses in ADNI. Acta Neuropathol. 121, 597–609. doi: 10.1007/s00401-011-0808-0, PMID: 21311900 PMC3175107

[ref22] Swardfager W. Lanctot K. Rothenburg L. Wong A. Cappell J. Herrmann N. (2010). A meta-analysis of cytokines in Alzheimer's disease. Biol. Psychiatry 68, 930–941. doi: 10.1016/j.biopsych.2010.06.012, PMID: 20692646

[ref23] Tarkowski E. Andreasen N. Tarkowski A. Blennow K. (2003). Intrathecal inflammation precedes development of Alzheimer's disease. J. Neurol. Neurosurg. Psychiatry 74, 1200–1205. doi: 10.1136/jnnp.74.9.1200, PMID: 12933918 PMC1738668

[ref24] Weiner M. W. Veitch D. P. Aisen P. S. Beckett L. A. Cairns N. J. Green R. C.. (2012). The Alzheimer's disease neuroimaging initiative: a review of papers published since its inception. Alzheimers Dement. 8, S1–S68. doi: 10.1016/j.jalz.2011.09.172, PMID: 22047634 PMC3329969

[ref25] Yang L. Lu R. Jiang L. Liu Z. Peng Y. (2009). Expression and genetic analysis of tumor necrosis factor-alpha (TNF-alpha) G-308A polymorphism in sporadic Alzheimer's disease in a southern China population. Brain Res. 1247, 178–181. doi: 10.1016/j.brainres.2008.10.019, PMID: 18992723

[ref26] Zhao A. Li Y. Deng Y. Alzheimer’s Disease Neuroimaging Initiative (2020). TNF receptors are associated with tau pathology and conversion to Alzheimer's dementia in subjects with mild cognitive impairment. Neurosci. Lett. 738:135392. doi: 10.1016/j.neulet.2020.135392 32947003

[ref27] Zhao J. O'Connor T. Vassar R. (2011). The contribution of activated astrocytes to Abeta production: implications for Alzheimer's disease pathogenesis. J. Neuroinflammation 8:150. doi: 10.1186/1742-2094-8-150, PMID: 22047170 PMC3216000

